# Pediatric Perineal Impalement Injuries: A Case Series on Surgical Management and Outcomes

**DOI:** 10.7759/cureus.91760

**Published:** 2025-09-07

**Authors:** Abdul Vakil, Rachith Sridhar, Majid Anwer, Abdul Hakeem S, Anurag Kumar, Farheen Ahmed

**Affiliations:** 1 Trauma & Emergency, All India Institute of Medical Sciences, Patna, Patna, IND; 2 Anesthesiology, All India Institute of Medical Sciences, Patna, Patna, IND

**Keywords:** case report, impalement, laparotomy, pediatric trauma, perineum

## Abstract

Falls represent a common cause of pediatric trauma, with perineal impalement injuries posing significant clinical challenges due to the anatomical complexity and involvement of multiple organ systems. In India, unsafe environments such as rooftops and construction sites contribute to the prevalence of such injuries among children, particularly boys. These injuries require prompt multidisciplinary management to reduce morbidity and mortality.

We present three pediatric cases of perineal impalement injuries caused by falls onto iron rods, managed at our level 1 trauma center. The patients, aged 8 to 10 years, sustained complex injuries involving the rectum, ileum, colon, mesentery, diaphragm, and liver. Management included exploratory laparotomy, controlled removal of the impaling object, primary repair of perforations, resection with stoma formation (ileostomy/colostomy), and intensive postoperative care. All patients experienced uneventful recoveries with successful stoma reversal and favorable long-term outcomes.

Pediatric perineal impalement injuries demand thorough clinical and radiological evaluation, often using CT imaging to assess internal damage. Surgical intervention requires careful extraction of foreign bodies, thorough debridement, and consideration of fecal diversion based on injury severity and contamination. A multidisciplinary approach involving pediatric surgeons, urologists, gynecologists, trauma surgeons, and radiologists is vital for optimal management. Early intervention and protocol-driven care minimize complications such as infections, fistulas, and sphincter dysfunction.

Although rare, pediatric perineal impalement injuries are life-threatening and complex. Timely diagnosis, meticulous surgical planning, and comprehensive postoperative management are essential for minimizing morbidity and preserving function. This case series underscores the critical role of a coordinated multidisciplinary approach in achieving successful outcomes in these challenging injuries.

## Introduction

Falls are identified as one of the most common mechanisms of injury in pediatric patients, particularly during play at home or in outdoor environments, contributing significantly to abdominal and pelvic trauma [1]. In the Indian context, Kundal et al. [2] found that children, particularly boys, are frequently exposed to unsafe settings such as rooftops or construction areas, leading to complex impalement injuries that often involve multiple organ systems. Pediatric perineal impalement injuries occur when a penetrating force causes penetration and retention of a long object into the perineal region, posing significant management challenges due to anatomical complexity [3]. These injuries present substantial clinical challenges due to the proximity of multiple vital organs such as the bladder, parts of the sigmoid colon, rectum, uterus, along with the fallopian tube and ovary in females, which have the potential for severe blood loss and the high risk of septic complications. Prompt and effective intervention, including accurate assessment and early multidisciplinary surgical management, is crucial in improving patient survival and minimizing long-term morbidity [4]. We report a case series involving three patients who presented with impalement injuries, managed successfully through timely surgical intervention at our level 1 trauma center.

## Case presentation

Case 1

**Figure 1:**
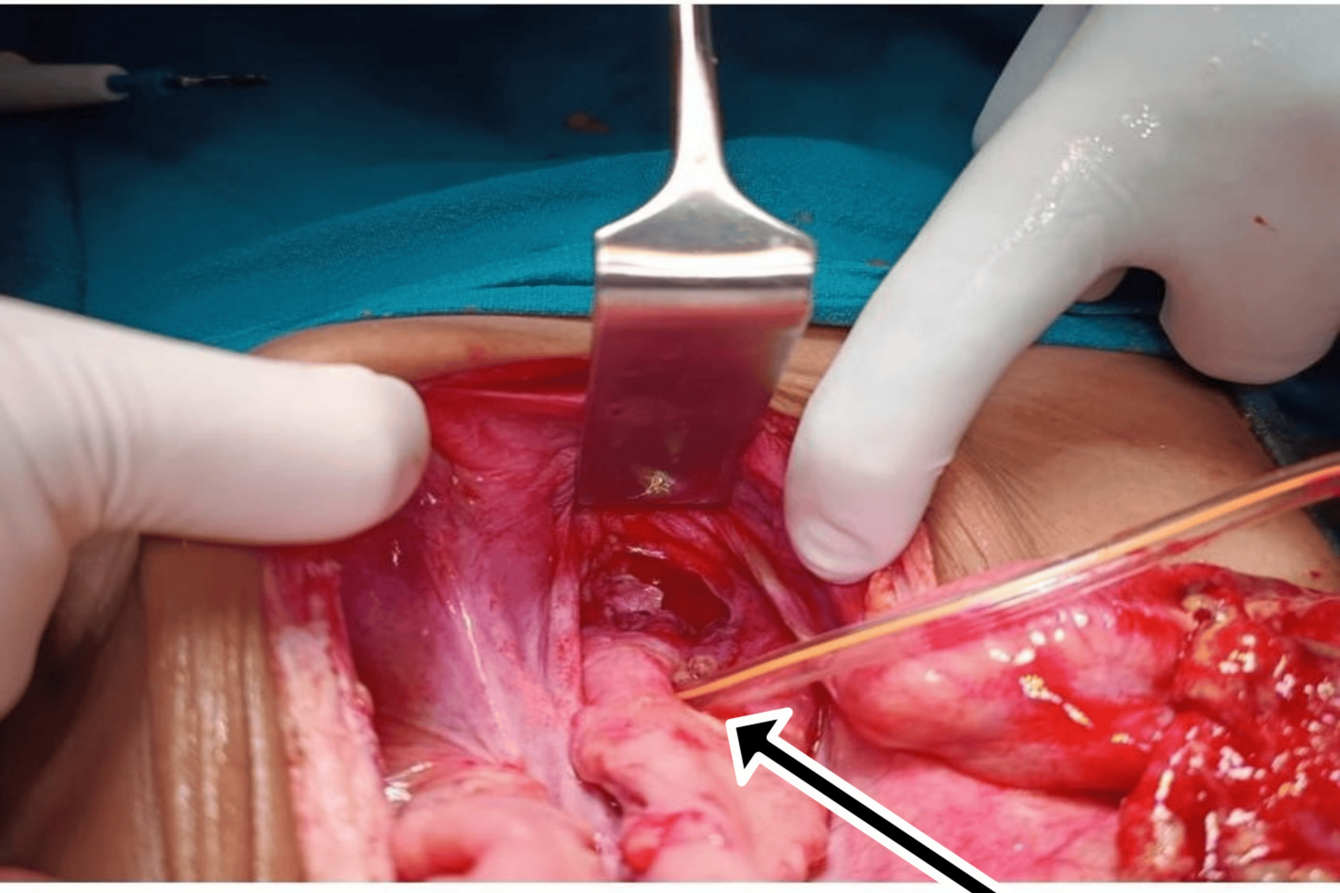
Intraoperative image showing anterior rectal wall tear

**Figure 2:**
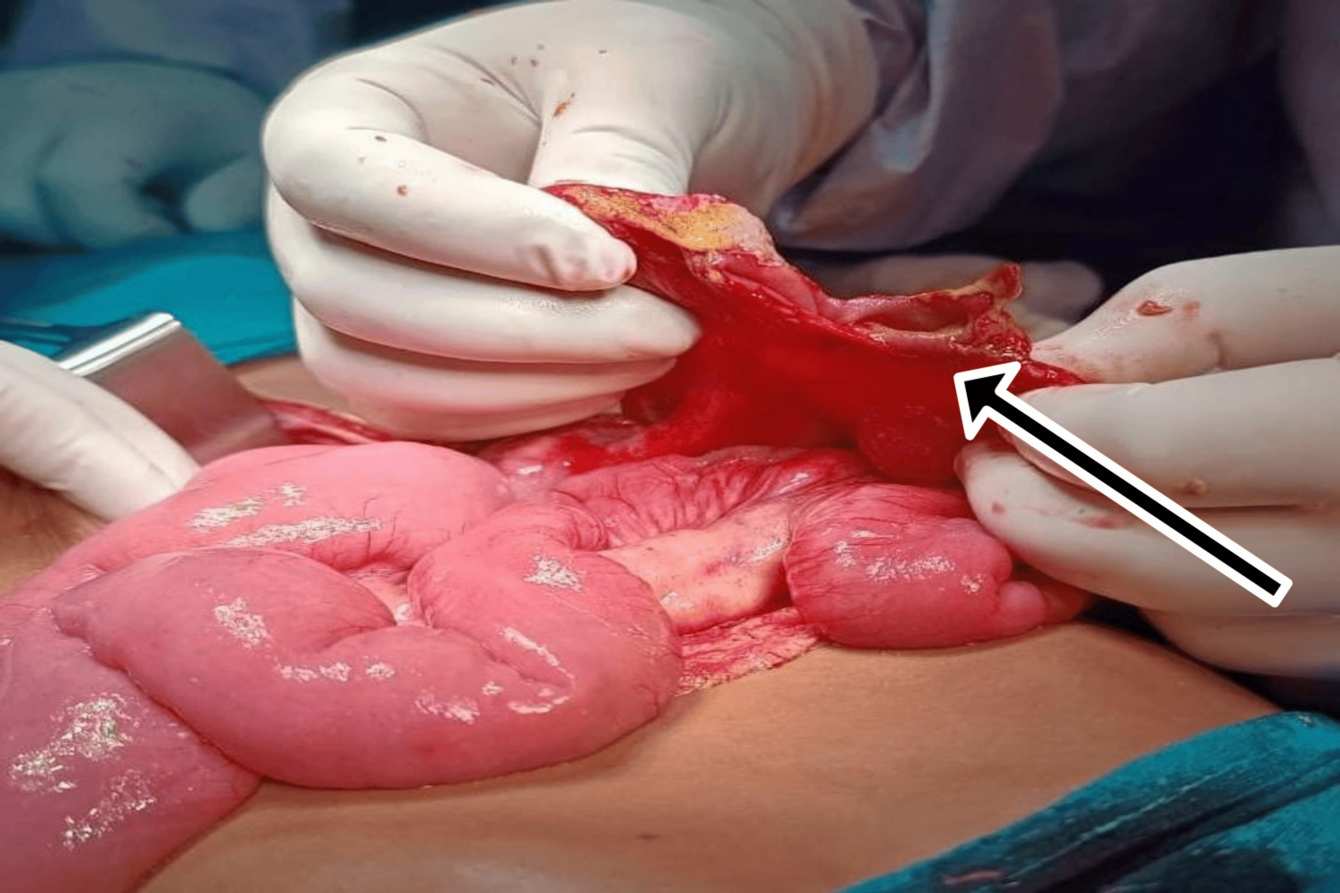
Intraoperative finding of circumferential tear of ileum

A 10-year-old male presented after falling from a height onto an iron rod, sustaining an impalement injury per rectum. The rod had been removed at a local hospital before referral. The patient arrived at our center approximately 28 hours after the injury. Primary survey was performed after completely exposing the child, which showed a perineal laceration of size 1x1 cm around the anus. There was no fecal contamination or per rectal bleed. Chest X-ray revealed air under the diaphragm. On the secondary survey, a tear in the anterior wall of the rectum was identified during a digital rectal examination. Per abdominal examination showed guarding and rigidity. After sending baseline lab parameters and blood for cross-matching, the patient was taken to the operating theater for an exploratory laparotomy. Intraoperatively, a 2 cm tear of the anterior rectal wall was found, along with a 2×1 cm perforation on the anterior surface of the rectosigmoid junction and a circumferential tear of the ileum approximately 20 cm proximal to the ileocecal junction (Figures 1, 2).

Surgical management included resection of a 10 cm perforated ileal segment, formation of a double-barrel ileostomy, and primary repair of both the rectal tear and rectosigmoid perforation. The patient was shifted to the ward postoperatively. He was started on intravenous fluids, broad-spectrum antibiotic ceftriaxone 500 mg IV bd for 5 days, and analgesics. The postoperative period was uneventful, and he was discharged on postoperative day 10 after suture removal. Stoma reversal was performed 3 months later. Patient was followed up after 1 and 3 months of discharge. He is doing well in follow-up.

Case 2

**Figure 3:**
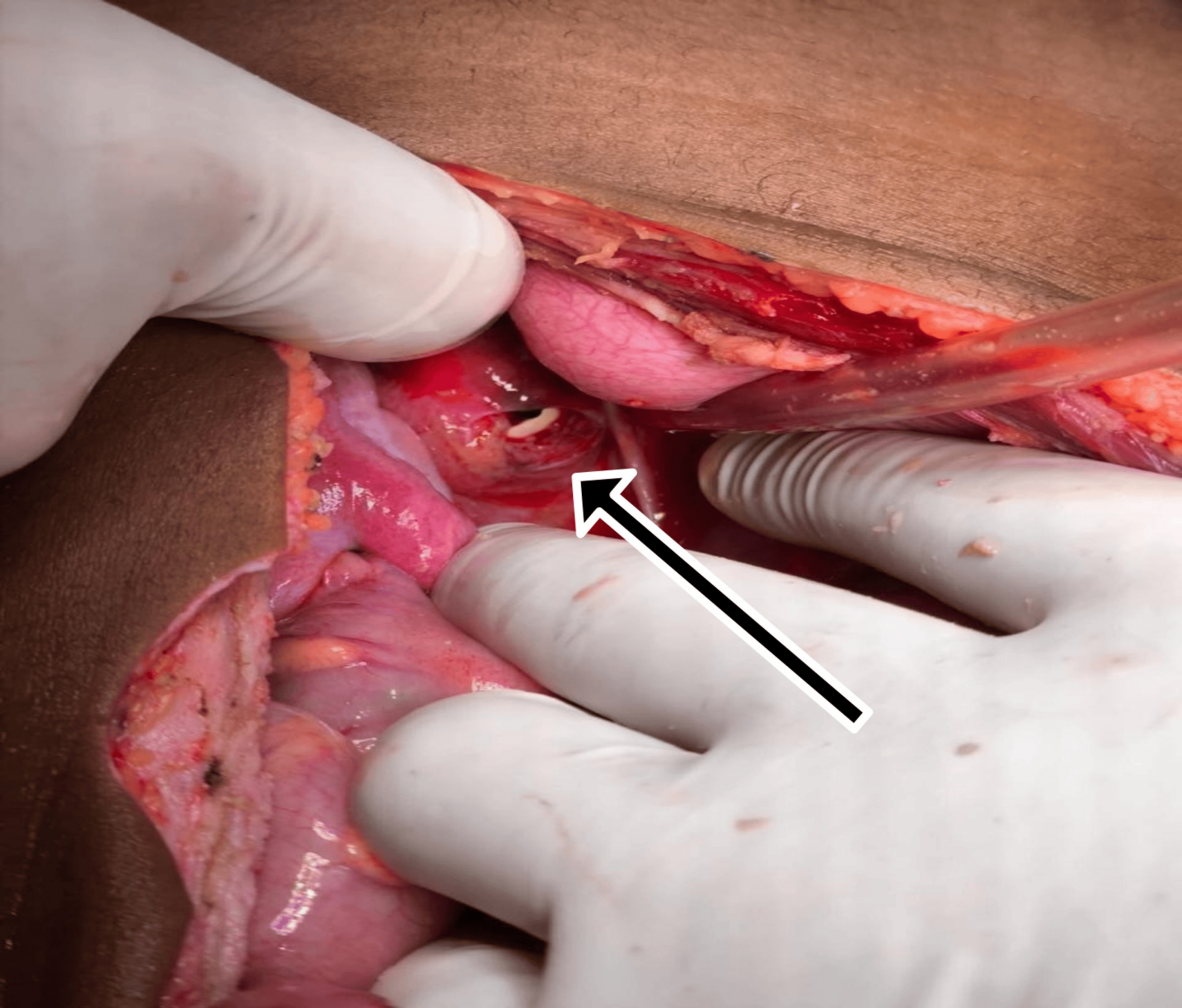
Intraoperative view of perforation at the upper third of the rectum

An 8-year-old female presented with a history of falling from a height of 10 feet onto an iron rod, resulting in an impalement injury per rectum. The impaled object had been removed at a local hospital. She was brought to our center after 24 hours of injury. On the primary survey, the airway was patent, and breathing was spontaneous. On exposing the child, there was a perineal laceration of size 2x1 cm with no fecal contamination. Per vaginal examination showed normal labia and clitoris. There was no per vaginal bleeding. Secondary survey showed diffuse guarding and rigidity in the abdomen. Her pulse rate was 140 bpm and blood pressure 100/87 mmHg. Adjuncts to the primary survey were within normal limits. In view of clinical signs of peritonitis, the patient was taken for urgent exploratory laparotomy. Intraoperatively, significant intraperitoneal fecal contamination was noted. A perforation measuring 1.5×1.5 cm was identified at the upper third of the rectum (Figure 3).

The surgical intervention included thorough peritoneal lavage, primary closure of the rectal perforation, and the creation of a diversion end sigmoid colostomy. The patient was shifted to the ward postoperatively. She was started on intravenous fluids, broad-spectrum antibiotic ceftriaxone 500 mg IV bd for 5 days, and analgesics. The postoperative period was uneventful, and she was discharged on postoperative day 10 after suture removal. Stoma reversal was performed 3 months later. She came for follow-up in OPD at 1 and 3 months of discharge. She was comfortable, taking food orally and passing feces and flatus regularly.

Case 3

**Figure 4:**
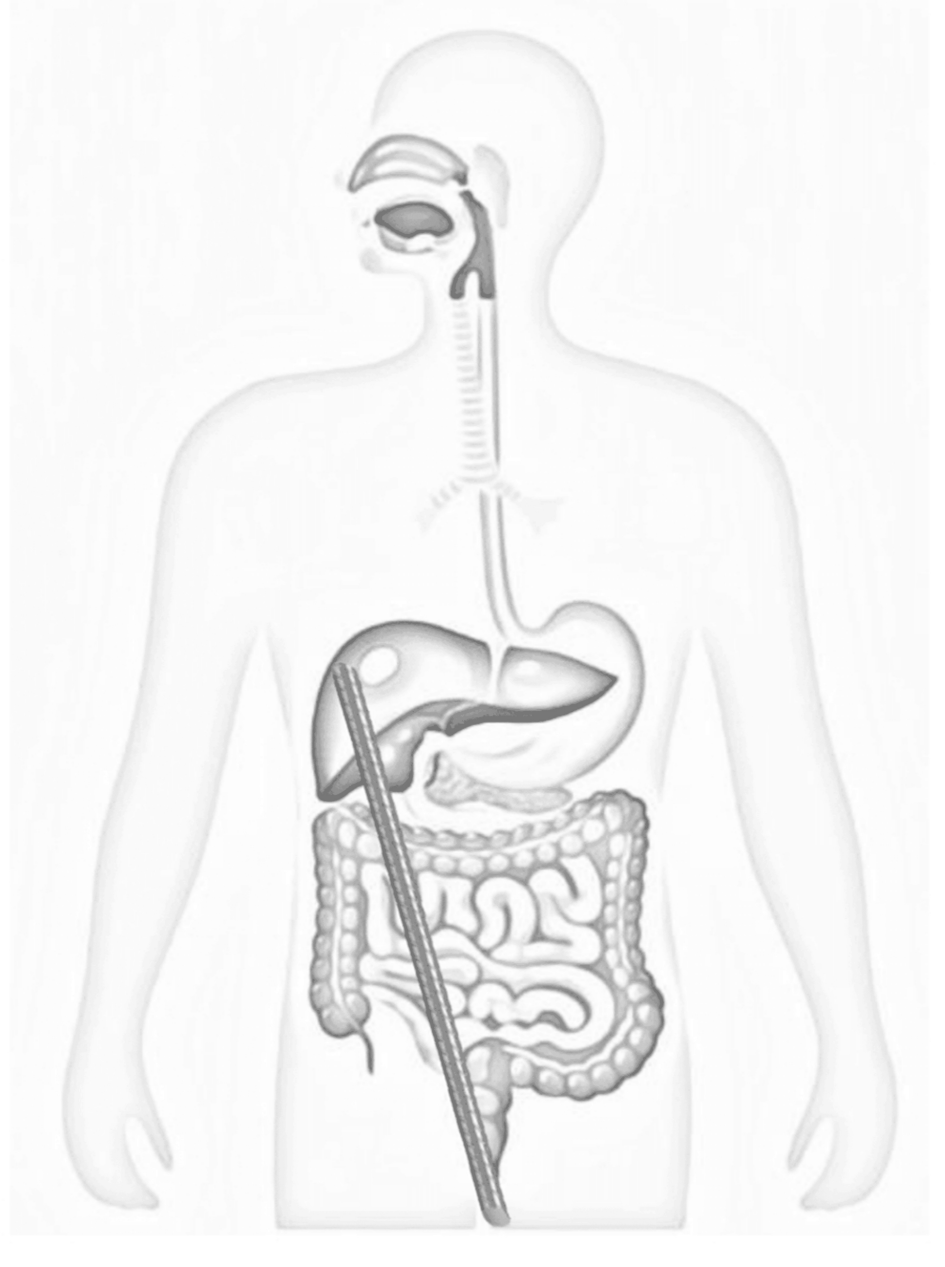
Figure showing the tract of the impaled object

**Figure 5:**
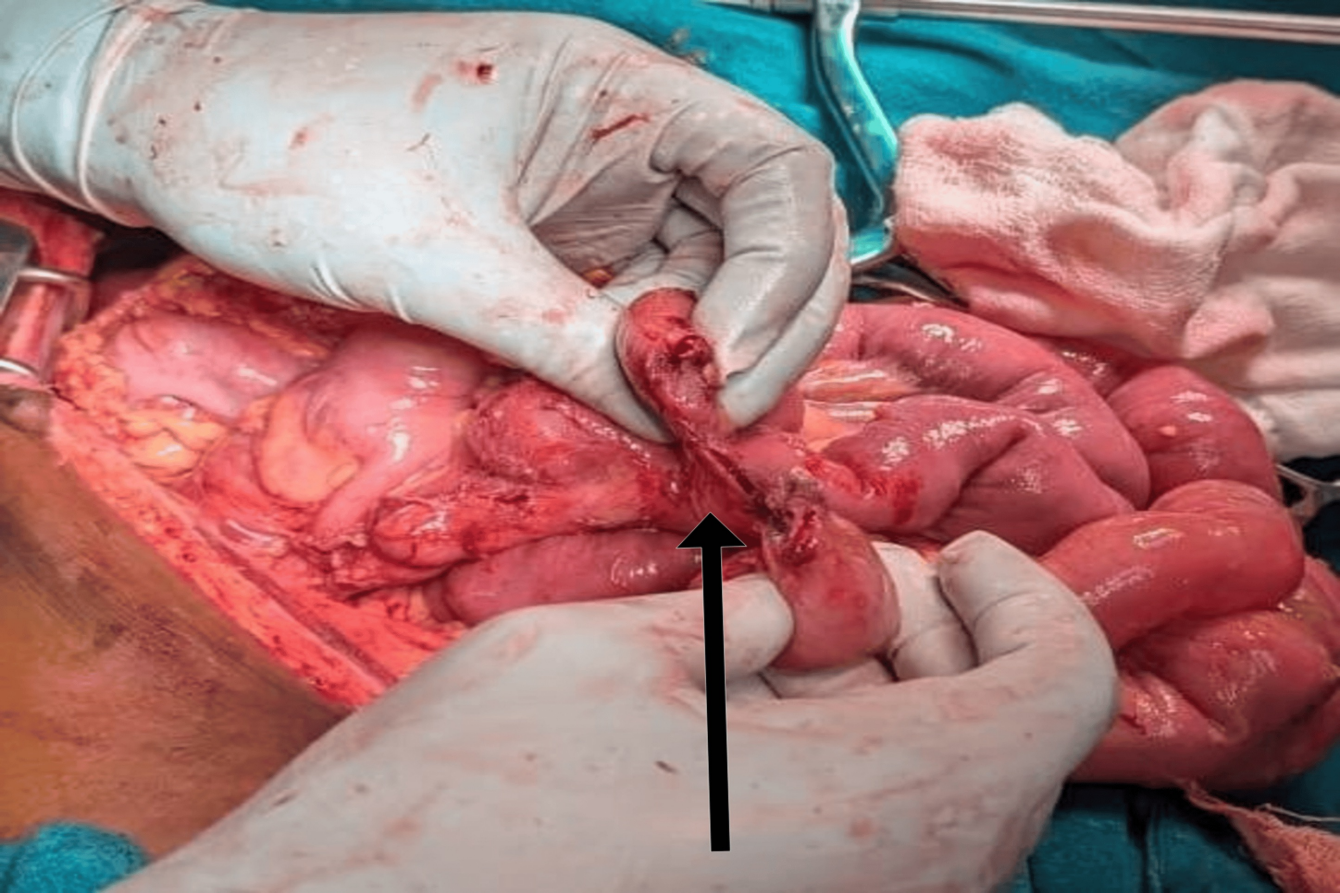
Intraoperative view showing a circumferential tear of the terminal ileum

A 10-year-old male presented following a fall from a height of 10 feet onto an iron rod, causing an impalement injury to the rectum. The patient arrived at our center within 30 minutes of sustaining the injury. On presentation, he had chest pain, tachypnea (respiratory rate = 28/min), hypotension (blood pressure = 80/60 mmHg), absent breath sounds on the right side, along with tracheal deviation to the left, suggestive of tension pneumothorax. Normal S1 and S2 heart sounds were present. Needle decompression was performed at the second intercostal space in the midclavicular line on the right side. Later on, an 18 F intercostal tube drainage was placed in the fifth intercostal space on the right side. The air column was moving. Perineal examination revealed the rod in situ. Per abdominal examination was deferred because the patient was experiencing significant pain, which would have made the examination difficult, potentially inaccurate, and possibly worsened his condition. The patient was immediately shifted to the operating theatre for exploratory laparotomy and removal of the impaled rod under direct vision. Intraoperatively, there was no significant contamination. The foreign body had entered via the upper third of the rectum, pierced the terminal ileum 10 cm from the ileocecal junction and mesentery, traversed the retroperitoneum, and again pierced the hepatic flexure of the colon, reaching the peritoneal cavity, the posterior aspect of segments V and VIII of the liver, and the right side of the diaphragm (Figures 4, 5).

**Figure 6:**
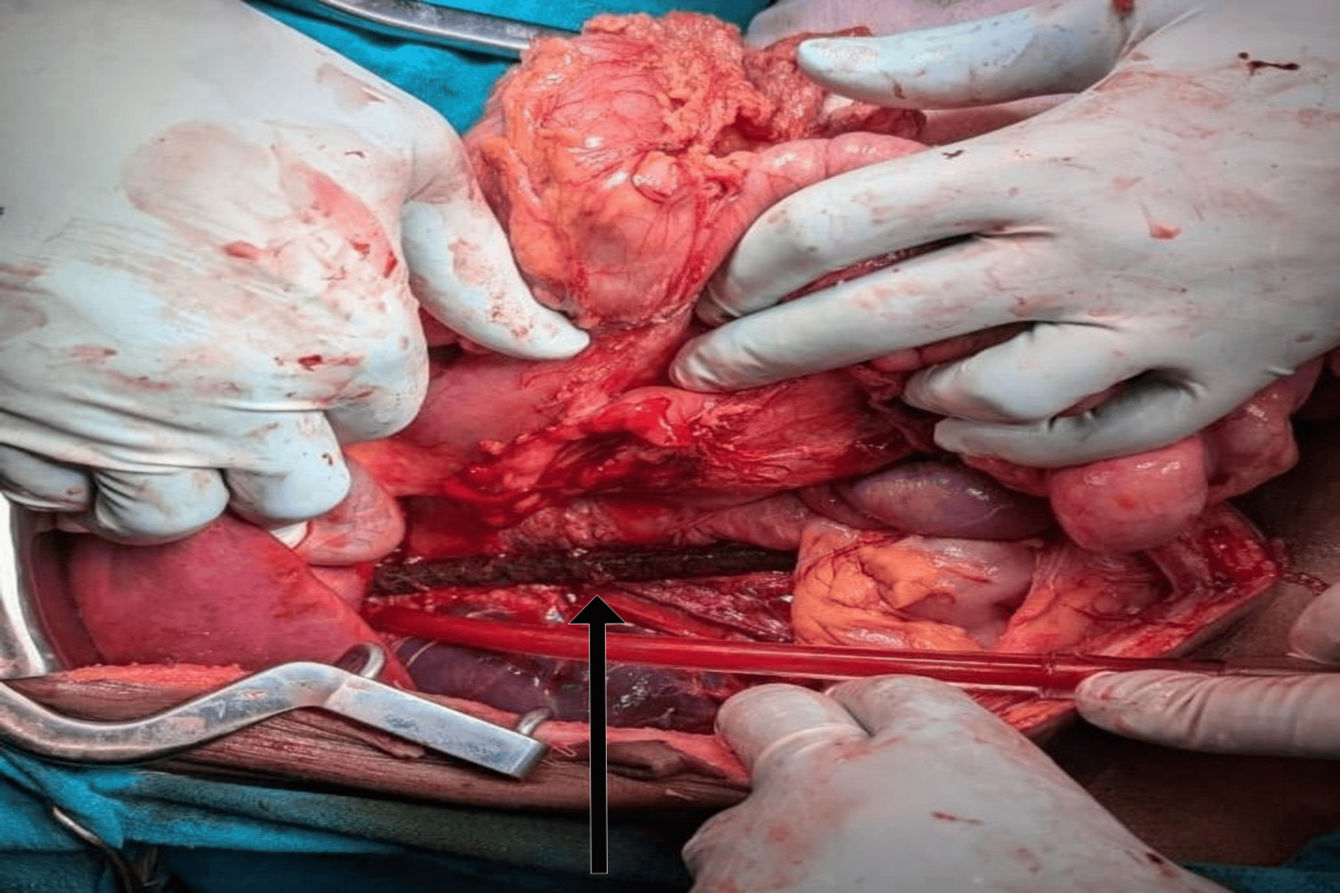
Cattell-Braasch manoeuver revealing the impaled rod lying parallel to the inferior vena cava

A Cattell-Braasch maneuver was performed to visualize the retroperitoneum. The rod ran parallel to the inferior vena cava without causing injury (Figure 6).

The rod was removed under direct vision. No additional hemothorax was noted. The diaphragmatic rent was repaired using horizontal mattress sutures. The perforations at the hepatic flexure and rectum were repaired primarily, and the terminal ileum was resected with the formation of a loop ileostomy. The abdominal wound was closed in layers. The patient was shifted to the ICU postoperatively. He was started on intravenous fluids, antibiotic ceftriaxone 500 mg IV BD, and analgesics. His postoperative course was uneventful. He was discharged on postoperative day 10 after suture removal. Stoma reversal was performed 3 months after the initial surgery. Patient came for follow-up at 1 and 3 months of stoma reversal. He is doing well, taking food orally and passing stool and flatus regularly.

## Discussion

A thorough clinical assessment of pediatric perineal impalement injuries involves careful inspection and evaluation under anesthesia due to the potential disparity between minimal external findings and severe internal injuries. CT of the abdomen and pelvis is considered the first-line diagnostic modality, facilitating the prompt identification and characterization of internal organ involvement and guiding the surgical management strategy effectively. Shrestha et al. [4], Anderson et al. [5], and Dreizin et al. [6] emphasized the role of CT in the evaluation of such penetrating injury. Surgical management of pediatric perineal impalement injuries requires meticulous wound care, thorough irrigation, and careful debridement of injured tissues, along with appropriate antibiotic therapy [7]. Intraoperative considerations include minimal manipulation of the impaling object until controlled conditions in the operating room are established, careful evaluation for hidden injuries through methods such as sigmoidoscopy, cystoscopy, or vaginoscopy, and determining the need for a fecal diversion based on hemodynamic status, injury severity, and contamination levels. A midline laparotomy is made, and an appropriate visceral rotation maneuver is performed along the tract. A proximal and distal vascular control is taken if the impaled object crosses a major vessel. The impaled object is then removed with simultaneous suction and packing. Torrential hemorrhage is expected after removal of the tamponade effect, which requires packing, clamping, or temporary ligation followed by repair. A search is then made of associated hollow viscus, solid organ, diaphragm, mesenteric, and retroperitoneal injuries.

A multidisciplinary team approach is essential in managing complex pediatric perineal impalement injuries, involving coordinated efforts from pediatric surgeons, urologists, gynecologists, trauma surgeons, and radiologists to optimize clinical outcomes. A pediatric surgeon is the overall leader of the resuscitation and surgical management in pediatric patients. Moreover, they can coordinate long-term follow-up for growth, continence, and quality of life. Trauma surgeons will help in advanced trauma life support-based resuscitation and in managing associated abdominal, thoracic, or vascular injuries intraoperatively, particularly in polytrauma victims. A urologist will help in evaluating urethral or bladder injuries (often with retrograde urethrogram or cystoscopy), and in managing urethral injury and establishing diversion (suprapubic catheter) when indicated. They will also contribute to long-term monitoring of urinary continence and voiding function. Intraoperative gynecologist involvement will ensure complete assessment of vaginal, uterine, or adnexal involvement in female patients. They will also help in repairing genital tract injuries with attention to preserving reproductive potential. A radiologist, by doing a contrast-enhanced CT abdomen, will help in delineating the injury trajectory, thus helping in choosing the most appropriate incision. A postoperative scan will help unravel the hidden cause of persistent fever, if any. The role of anesthesiologists and critical care specialists cannot be underemphasized. They provide safe anesthesia for prolonged procedures and manage hemodynamics in unstable patients. They also oversee postoperative critical care, pain management, and ventilatory support when indicated. Effective communication among specialists facilitates comprehensive injury assessment, timely surgical intervention, and postoperative care, thus significantly reducing morbidity and improving long-term functional recovery. Surgical management of pediatric perineal impalement injuries involves initial careful assessment under anesthesia, followed by meticulous exploratory laparotomy to identify and repair injuries to abdominal, pelvic, and possibly thoracic organs [8]. Techniques include controlled extraction of impaled objects under direct visualization, appropriate fecal diversion via colostomy, ileostomy for significant colorectal injuries, and primary repair of affected structures such as the bladder, bowel, diaphragm, and liver, often necessitating a multidisciplinary surgical team [9]. Traditionally, routine fecal diversion with colostomy/ileostomy was performed for all penetrating colon and rectal injuries to reduce pelvic sepsis and mortality. Primary repair or resection with anastomosis is now safe in most intraperitoneal colorectal injuries with the advancement in antibiotics and critical care. The indication for fecal diversion can be divided into patient-, injury-, or site-related factors. Patients with hemodynamic instability, polytrauma, malnutrition, and immunosuppression can be diverted. Delayed presentation (more than 24 hours) with gross fecal contamination is also best considered for diversion with a few exceptions. Bosarge et al. advocated that extraperitoneal rectal injuries should ideally be diverted after repair [10]. Selected isolated rectal or colonic injuries can undergo primary repair without diversion [11]. In our case series, patients underwent primary repair along with fecal diversion, given delayed presentation with gross fecal peritonitis and associated injuries. Common complications following pediatric perineal impalement injuries include wound infections, anal or vaginal stenosis, fistula formation, and temporary or permanent anal sphincter insufficiency. Effective postoperative care involves meticulous wound management, fecal diversion through colostomy for extensive colorectal injuries, and timely identification and management of complications such as strictures and fistulas, often requiring conservative methods or surgical revision. The management of urinary diversion for urethral/bladder injuries requires a delayed repair. Involvement of the anal sphincter will lead to fecal incontinence. Such patients undergo detailed evaluation, including anal manometry and repair, before stoma reversal. Sugar et al. [12] also emphasized the possibility of child abuse in patients with perineal injury. A detailed history taking, physical examination (often under anesthesia), and collaboration with child protection teams are essential. Key red flags include inconsistent history, delayed care, or injury patterns inconsistent with the reported mechanism.

**Figure 7:**
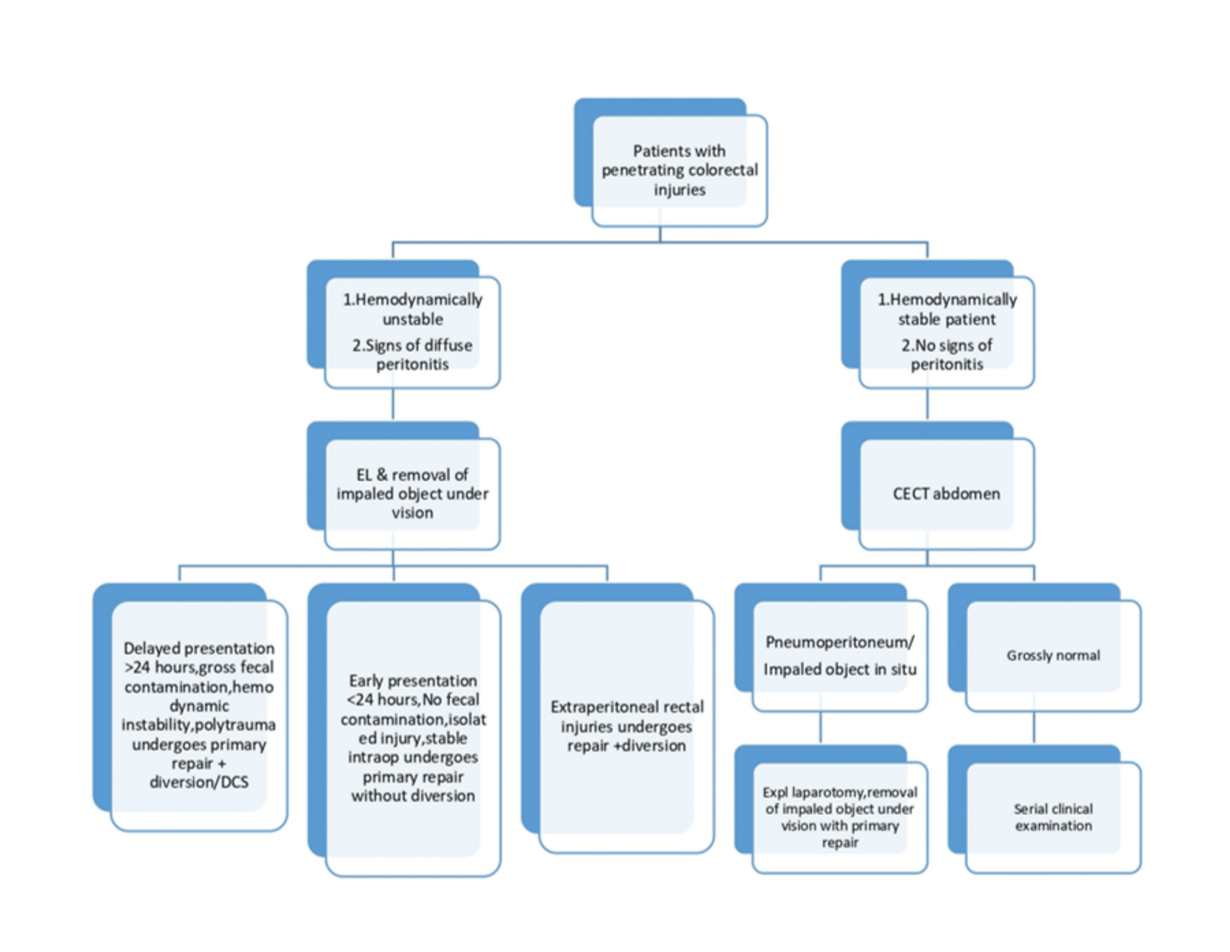
Protocolized approach in patients with penetrating colorectal injuries at our center CECT, Contrast-enhanced computed tomography; DCS, Damage control surgery; EL, Exploratory laparotomy; Expl, Exploratory

The prognosis for pediatric perineal impalement injuries is generally favorable, especially when managed with timely surgical intervention, appropriate primary repair with or without diversion, and careful postoperative monitoring. Key prognostic factors influencing outcomes include the severity and extent of injury, the presence of associated organ damage, early diagnosis, and adherence to a multidisciplinary and protocol-driven management approach, which significantly reduces morbidity and preserves functional integrity [8,12]. A protocolized approach for penetrating perineal injuries ensures uniform evaluation, minimizes missed injuries, and improves outcomes (Figure 7) [13].

## Conclusions

Pediatric perineal impalement injuries, though rare, are potentially life-threatening due to their anatomical complexity and risk of multisystem involvement. Timely diagnosis, careful imaging, and a multidisciplinary surgical approach are critical for optimal outcomes. The successful management of the cases presented in this series highlights the importance of individualized surgical planning, prompt intervention, and structured postoperative care in minimizing morbidity and preserving function.
